# Cellular Immune Responses in Rainbow Trout (*Onchorhynchus mykiss*) Following Vaccination and Challenge Against Salmonid Alphavirus (SAV)

**DOI:** 10.3390/vaccines8040725

**Published:** 2020-12-02

**Authors:** Kimberly A. Veenstra, Kjartan Hodneland, Susanne Fischer, Kota Takehana, Rodrigo Belmonte, Uwe Fischer

**Affiliations:** 1 Friedrich-Loeffler-Institut (FLI), Federal Research Institute for Animal Health, Institute of Infectology, Südufer 10, 17493 Greifswald-Insel Riems, Germany; kimberly.veenstra@fli.de (K.A.V.); susanne.fischer@fli.de (S.F.); 2 MSD Animal Health Innovation, Thormøhlens Gate 55, 5006 Bergen, Norway; kjartan.hodneland@merck.com (K.H.); rodrigo.belmonte@merck.com (R.B.); 3 Nagano Prefectural Fisheries Experimental Station, 2871 Oaza-Nakagawate, Akashina, Azumino-shi, Nagano 399-7102, Japan; takehana-kota-r@pref.nagano.lg.jp

**Keywords:** salmonid alphavirus (SAV), cell-mediated immune response, pancreas disease, cytotoxicity, rainbow trout, ELISA, gene expression, adjuvant, vaccine

## Abstract

Viral disease outbreaks remain a significant limiting factor for aquaculture. The majority of licensed vaccines used in the industry are administered as oil-adjuvanted formulations carrying inactivated whole pathogens. Cell-mediated immune responses, in particular those based on virus-specific cytotoxic T-cells (CTLs) to conventional inactivated oil-based vaccines, are largely unexplored. As vaccines cannot be optimized against viral pathogens if knowledge of host cellular immune mechanisms remains unknown, in this study we examined fundamental cell-mediated immune responses after vaccination of rainbow trout with an oil-adjuvanted inactivated vaccine against salmonid alphavirus (SAV) and after infection with SAV. A unique *in vitro* model system was developed to examine MHC class I restricted CTL responses in a clonal line of rainbow trout. The levels of cell-mediated cytotoxicity were compared to pathology, virus load, specific antibody response, changes in immune cell populations, and mRNA expression. Our results hint that different protective mechanisms are being triggered by infection compared to vaccination. While vaccination itself did not cause a strong cytotoxic or humoral response, subsequent challenge of vaccinated fish resulted in significantly stronger and faster specific cytotoxicity, alongside reduced viral titers and pathology. Hence, testing a vaccine on the capacity to induce cell-mediated cytotoxicity will still require a challenge test. Examination of cellular markers additionally indicates that the initial innate response induced by the vaccine could play an important role in steering adaptive mechanisms.

## 1. Introduction

Intensive fish farming is a major global industry. Disease outbreaks remain a significant limiting factor and are considered the single largest cause of economic loss [1,2]. Despite fish having immune mechanisms comparable to higher vertebrates [3,4], farmed fish often fail to mount optimal anti-viral responses and are at high risk from emerging viral diseases [5,6]. Due to limited therapeutic measures, vaccination is the only effective strategy for preventing diseases in aquaculture [7]. The majority of licensed vaccines are administered as oil-adjuvanted formulations carrying inactivated whole pathogens [8]. These inactivated vaccines are successful in inducing immunity against extra-cellular pathogens such as bacteria [9]; but they are often sub-optimal against some viruses. The lack of a cell-mediated response to these types of vaccines, particularly the expansion of virus-specific cytotoxic T lymphocytes (CTLs), could be a reason for this sub-optimal efficacy [10].

Cell-mediated cytotoxicity (CMC) is a mechanism in which host immune cells recognize and kill virus-infected cells, and is critical for anti-viral immunity in mammals and teleosts. The main cytotoxic cells are natural killer (NK) cells and cytotoxic T-lymphocytes (CTLs) belonging to the innate and the adaptive branches of the immune system, respectively. While NK cells can immediately and spontaneously recognize virus-infected cells, CTLs require MHC class I restricted induction to effectively eliminate viral infections [11,12,13]. In short, as viruses replicate intra-cellularly they force host cells to assemble and express viral proteins. These infected cells will subsequently present the viral peptides loaded onto MHC class I molecules on their surface. CD8^+^ CTLs will recognize these peptides via their T-cell receptor complex, which triggers the destruction of the infected host cell [13].

To date, there has been no comprehensive study of virus-specific CMC responses in fish following the injection of inactivated oil-adjuvanted viral vaccines. To expand current knowledge on vaccine induced cellular immune responses and to elucidate mechanisms contributing to host protection, we established a unique *in vitro* model system based on previous work by [14] and [15]. This assay utilizes effector cells from a clonal line of rainbow trout and infectible MHC class I matched and mismatched target cell lines to examine CTL responses following vaccination and infection.

Salmonid alphavirus (SAV) is an RNA virus in the family *Togaviridae*, genus *Alphavirus* and causes pancreas disease in Atlantic salmon (*Salmo salar*) and sleeping disease in rainbow trout (*Oncorhynchus mykiss*), and was used as a model pathogen in this work. Pancreas disease is of major concern in Atlantic salmon farming in Europe, associated with frequent and high mortality rates (up to 60%) and reduced weight gain afterwards [5,10]. Commercially available inactivated vaccines against SAV have been found to be efficacious following injection as a monovalent or polyvalent vaccine [16,17,18,19]. However, despite comprehensive vaccination programs, SAV is becoming increasingly prevalent in the field and the eradication of this pathogen is a challenge for most fish farms [10].

Therefore, in this paper the immune mechanisms after vaccination with an oil adjuvanted inactivated vaccine against pancreas disease were investigated. Pathology, virus loads, CMC, specific antibody responses, and changes in immune cell populations in rainbow trout were compared after administration of: (a) A monovalent oil-adjuvanted vaccine, (b) the corresponding adjuvant, (c) live SAV, and (d) live SAV challenge to fish which had been vaccinated. These experiments suggest that the mechanisms induced by SAV infection and vaccination are different. While vaccination itself did not trigger a strong specific cytotoxic response, a significant and more rapid induction of virus specific cytotoxicity following infection, along with reduced viral titers and pathology has been recorded. Consequently, careful evaluation of the capacities of a vaccine to induce cell-mediated immune responses still requires viral challenge. Our data also suggests that the innate response triggered by adjuvants is an additional, important component of vaccine-induced protection against SAV.

## 2. Materials and Methods

### 2.1. Fish

Homozygous isogenic rainbow trout clone C25 expressing the MHC class I allele Onmy-UBA*501 were derived from Nagano Prefectural Experimental Station of Fisheries (Akashina, Nagano, Japan). The clone was produced by gynogenesis over two generations by suppression of mitosis and meiosis in the first and second generations, respectively. Clonality was confirmed by DNA fingerprinting (unpublished data). Fish were kept at 12–13 °C in aerated water tanks and fed commercial dry pellets. Fish had an average body weight of 297 (±72) g at the beginning of the study.

### 2.2. Treatment Groups

Four treatment groups were examined during this study to compare immune responses as described in Table 1. Naïve, untreated fish were sampled prior to the start of the experiments (0 dpv/dpi) and are considered “control fish” for all groups. Groups 1, 2, and 4 were naïve fish prior to injection with adjuvant, vaccine or virus; Group 3 had been vaccinated 43 days prior to infection with live SAV. Inactivated oil adjuvanted vaccines are thought to confer protection in salmonids by 550 degree days, which corresponded to 6 weeks (43 days) post injection in this study.

### 2.3. Vaccination

The test vaccine (inactivated SAV oil-adjuvanted emulsion) was provided by MSD Animal Health Innovation (Bergen, Norway) as well as the control treatment, a placebo vaccine that was emulsified without antigen and hereafter referred to as “adjuvant”. For vaccination, fish were anaesthetized in benzocaine solution (10 mg/L rearing water) and intraperitoneally (i.p.) vaccinated with 0.1 mL of the test vaccine or adjuvant.

### 2.4. Challenge

For the production of challenge virus, confluent CHSE cells (cell-line information and maintenance protocol described in Section 2.7.2) were split at a ratio of 1:3 into T75 flasks (Corning, Taufkirchen, Germany) in mixed medium (MM) (Iscove’s MDM/Ham’s F12 (Gibco, Schwerte, Germay) at a ratio of 1:1), supplemented with 10% fetal bovine serum (FBS) and incubated overnight at 20 °C, 2.5% CO_2_. The next day 500 µL of SAV subtype 3 (SAV3) supernatant was added to the cells and the flasks incubated at 15 °C, 2.5% CO_2_ until 80% cytopathic effect (CPE) was observed. The flasks were freeze-thawed, after which the resulting suspension was pipetted vigorously to ensure complete cell destruction. This suspension was centrifuged (450 g, 10 min, 4 °C) and the cleared supernatant collected, aliquoted and stored at −80 °C. Virus titers were determined by adding a log_10_ serial dilution of the virus supernatants to CHSE cells and by observing CPE over time. 10^2.05^ TCID_50_ of SAV3 in 50 µL were injected intramuscularly (i.m.) into the dorsal muscle of anesthetized fish 43 dpv.

### 2.5. Virus Load

Blood was collected from the caudal vein of anaesthetized fish and placed in BD Microtainer^®^ SST™ tubes, allowed to clot for 30 min at room temperature, and centrifuged (8000× *g*, 2 min). Sera were collected, aliquoted and stored at −80 °C until use. RNA from serum samples was extracted using an RNeasy^®^ 96 Kit (Qiagen, Oslo, Norway) according to the manufacturer’s instructions (elution volume: 50 μL per RNA sample). RT-qPCR analyses were performed with a Verso™ 1-step RT-qPCR Low-ROX Kit (Thermo Scientific, Oslo, Norway) using 4 μL of RNA per sample. Primer and probe concentrations for nsP1 assay [20] were: Fwd. primer: 900 nM, Reverse primer: 900 nM, Probe: 260 nM. Reactions were run in an ABI PRISM^®^ 7500 FAST Thermocycler from Applied Biosystems (AB) at the following conditions: 50 °C for 30 min; 95 °C for 15 min; 40 cycles of: 95 °C for 15 s, and 60 °C for 60 s.

### 2.6. Pathology

Heart and pancreas were excised and placed in 4% neutral buffered formalin (Carl Roth, Karlsruhe, Germany). Sections for histopathology were processed by standard paraffin wax techniques and stained with hematoxylin and eosin (H&E). Tissue sections were examined as a blind study and the presence or absence of lesions recorded. A scoring system was used to evaluate the severity of SAV induced lesions in heart and exocrine pancreas [21].

### 2.7. Determination of Cell-Mediated Cytotoxicity (CMC) against SAV Infected Cells

#### 2.7.1. Lactate Dehydrogenase (LDH)-Based Cytotoxicity Assay

The LDH assay was performed using a commercially available LDH Cytotoxicity Detection Kit (Takara, Göteborg, Sweden) as per the manufacturer’s instructions, and as described by [15]. Briefly, varying numbers of effector cells from fish treated as described in Table 1 were added to a constant number of non-infected or SAV infected MHC-I matched or MHC-I mismatched target cells (E:T). Confirmation that the target cells had a matched or mismatched MHC-I molecule with the C25 clonal fish used in these *in vivo* experiments was done by RT-qPCR applying primers that specifically amplify the Onmy-UBA*501 gene as described previously [22]. The effector and target cells were incubated together at 15 °C for 4 h in 96-well flat bottom cell culture plates (Greiner, Kremsmünster, Austria), in triplicate. After the incubation period, the plates were centrifuged (10 min, 250× *g*, 4 °C) and 100 µL of supernatant was removed and added to new 96-well flat bottom plates (Greiner). 100 µL of cytotoxicity assay substrate was then added and the plates were left to develop at room temperature for 30 min. Plate absorbance was read in a Spectra max Plus (Analytical Technologies) plate reader at 490 nm (ref 655 nm) using Softmax Pro 5.3 software. A detailed description of background controls and the formula used to calculate cytotoxicity can be found in Appendix A.

#### 2.7.2. Preparation of Target Cells

MHC-I matched target cells were RTG-2/38 (rainbow trout gonad [ATCC]/Bank for Cell Lines in Veterinary Medicine, FLI, Insel Riems, Germany) (hereafter referred to as RTG2)). The MHC-I mismatched target cells were CHSE/1104 (Chinook salmon embryo [ECACC]/Bank for Cell Lines in Veterinary Medicine, FLI, Insel Riems, Germany) (hereafter referred to as CHSE)). These cells were maintained in MM + 20% FBS at 20 °C, 2.5% CO_2_. Prior to the CMC assay, RTG2 and CHSE cells (4 × 10^4^ cells/well) were seeded into 96-well flat bottom cell culture plates (Greiner) in MM + 10% FBS. Immediately after seeding, SAV subtype 1 (SAV1) was added to the target cells at an MOI (multiplicity of infection) of 50, which had been previously determined by immunofluorescence staining of SAV1 to yield an initial infection of at least 80% of cells. After 48 h incubation at 15 °C, the wells were washed 3× using serum-free MM. 100 µL of serum-free MM supplemented with 0.1% bovine serum albumin (BSA) (Sigma-Aldrich, Steinheim, Germany); insulin-transferrin, sodium selenite (ITS) (Sigma-Aldrich); 100 U/mL Penicillin and 100 µg/mL Streptomycin (P/S) (Genaxxon, Münster, Germany)) was then added to each well. The effector cells (isolated as described in Section 2.7.3) were then added to each well.

#### 2.7.3. Preparation of Effector Cells

Fish were anaesthetized and blood was collected from the caudal vein into a syringe rinsed with heparin (Sigma-Aldrich) (1000 U/mL in phosphate buffered saline (PBS)). Spleens were then removed and placed in beakers containing MM supplemented with 20% FBS and P/S. Small pieces were individually separated from each spleen for further RT-qPCR analysis and the remaining spleen tissues from the respective sampling groups were pooled to prepare single cell suspensions for flow cytometry analysis and for CMC assays. Effector cells were isolated from pooled spleens and individual blood samples using the hypotonic method as previously described [23]. After the final centrifugation, PBLs from each fish were individually stored for RT-qPCR analysis, and the remaining cells were then pooled for CMC assay and flow cytometry analysis. This took advantage of using clonal fish, where pooling of leukocytes does not result in adverse effects and ensures sufficient numbers of effector cells. The splenocytes and PBLs were resuspended in MM + 20% FBS + P/S and placed in a T75 cell culture flask (Corning) and incubated at 15 °C overnight. The following day, cells were removed from the flasks (with adhesive cells removed via gentle scraping using 0.5 mM EDTA in PBS, and viable lymphocyte-like cells were counted using a Thoma haemocytometer and trypan blue (Sigma-Aldrich) exclusion to calculate the effector to target cell ratios. The effector cells were washed and resuspended at the required dilution in serum-free supplemented MM. One hundred microliters of effector cells in supplemented MM were then added to the wells containing SAV infected and non-infected target cells at a range of E:T ratios from 1:100 to 1:12.5.

### 2.8. Flow Cytometry

Pooled splenocytes and PBLs were isolated as described in Section 2.7.3 and processed for flow cytometry analysis as follows. A total of 2 × 10^5^ cells per sample and well were added to a round-bottom 96-well plate (Greiner). The cells were then pelleted by centrifugation at 250× *g* for 3 min and the media discarded. The cell pellets were resuspended in 50 μL primary antibody diluted in MM, as described in Table 2.

The cells were incubated for 30 min at 4 °C and washed once. Cells were gently resuspended in 50 µL of secondary antibody diluted in MM (as described in Table 2) and incubated for 30 min at 4 °C. Cells were washed once and resuspended in 200 µL of MM prior to analysis.

Control cells were treated only with the corresponding conjugated secondary antibody. Dead cells were excluded by DAPI staining considering DAPI^-^ cells only. For specific staining with monoclonal antibodies (see Table 2) only FSC^low^/SSC^low^ (lymphocyte-like) cells were considered. A BD FACS CANTO II (BD Biosciences) was used to analyze the samples, and at least 10,000 events were recorded for each sample. Analysis was performed using BD FACSDiva Software (v8.0.1).

### 2.9. Tissue Sampling, RNA Extraction, cDNA Synthesis and Reverse Transcription Quantitative Polymerase Chain Reaction (RT-qPCR)

At the time points described in Table 1, 100 mg of spleen tissue was placed in 2 mL Eppendorf tubes containing 350 µL of lysis buffer (Macherey-Nagel, Düren, Germany) with a 5 mm stainless steel bead and homogenized for 2 min at 30 Hz in a Qiagen TissueLyser II before being stored at −20 °C until processing. Following cell isolation described in Section 2.7.3 PBLs (approx. 5 × 10^7^ cells) from individual fish were placed in 1.5 mL Eppendorf tubes, centrifuged (500× *g*, 5 min, 4 °C), then resuspended in 350 µL of lysis buffer (Macherey-Nagel) and homogenized for 2 min at 30 Hz before being stored at −20 °C until processing. RNA was extracted using a NucleoSpin RNA II total RNA extraction kit (Macherey-Nagel) as per the manufacturer’s instructions. RT-qPCR was performed using a SensiFAST™ SYBR^®^ No-Rox One-Step Kit (BioLine, Heidleberg, Germany) in 96-well transparent plates (Kisker Biotech, Steinfurt, Germany) as per the manufacturer’s instructions with a MX3000P cycler and software. A 3-step cycling protocol was used as follows: 45 °C for 10 min followed by 95 °C for 2 min; 40 cycles of: 95 °C for 5 s; 60 °C for 10 s, 72 °C for 15 s. Quantification cycles (Cq) for the genes examined were normalized to the housekeeping gene elongation-factor 1α (EF-1α). The EF-1α gene was used as a house keeping gene in this study as it has been found to be the most suitable for gene expression studies in salmonids [27].

#### Immune Genes

Genes relating to the pro-inflammatory (IL-1β), anti-viral (toll-like receptor-(TLR)-3, Mx, Viperin), Th1 (IFN-γ) and Th2 (IL-4/13) responses as well as genes associated with cell populations (natural killer cells (Natural Killer Cell Enhancement Factor-NKEF), macrophages (Macrophage Colony Stimulating Factor Receptor-MCSFR), dendritic cells (C-type lectin-CLEC4T1/CD209), cytotoxic cells (perforin (PFN)-1) and MHC-I and -II were selected to be examined for changes in expression at 3, 7, 14, 21, and 28 days post-vaccination and post-challenge in spleen tissue and PBLs. The specific forward and reverse primers used for each gene and other associated information can be found in the Supplementary Materials (Table S1). Primer efficiency was determined to be between 90–110% for all genes.

### 2.10. ELISA

Sera were collected from vaccinated and challenged fish 63 dpv/dpi (*n* = 12/group) as described in Section 2.5.

For the preparation of the coating antigen an SAV1 viral suspension was produced as described in Section 2.4 and then concentrated by iodixanol gradient (Progen) ultra-centrifugation, as described by [28]. Protein concentration was determined using a Pierce™ BCA Protein Assay Kit (Thermo Scientific, Dreieich, Germany). The concentrated SAV antigen was diluted to 10 μg/mL in carbonate bicarbonate buffer, pH 9.6 (Sigma-Aldrich). Fifty microliters of coating antigen was added to each well of a 96-well Polysorp NUNC F plate (Thermo Fisher, Darmstadt, Germany) and left to incubate overnight at 4 °C. The plate was washed 3× with washing buffer (PBS with 0.1% Tween20 (PBST)) and 100 µL of 1× ROTI^®^ BLOCK (Carl Roth) reagent was added to each well and left to incubate at room temperature for 1 h. The plate was then washed 3× and 50 µL of sera diluted 1:100 in washing buffer were added to each well. The plate was left to incubate at room temperature for 2 h while shaking (250 rpm) on an orbital shaker. The plate was washed 3× and 50 µL of mouse anti-salmonid Ig (clone 5F12) (Bio-Rad AbD Serotec, Puchheim, Germany) (1:500 in washing buffer) was added. The plate was incubated at room temperature for another hour while shaking (250 rpm). The plate was washed 3× and 50 µL of Pierce™ goat anti-mouse IgG, IgM (H + L) HRP conjugate (Thermo Fisher) (1:10,000 in washing buffer) and left to incubate for 1 h at room temperature while shaking (250 rpm). The plate was washed 3× and 50 µL of 1-Step Ultra TMB ELISA (ThermoScientific) was added to each well and left to incubate at room temperature in the dark for 15 min. 25 µL of stop solution (2N H_2_SO_4_) was added, and the plate was read in a Spectra max Plus spectrophotometer using Softmax Pro 5.3 Plus software (ELISA endpoint). (NOTE: Cross-reactivity testing using sera from rainbow trout challenged with SAV subtypes 1, 2 or 3 against coating antigen of SAV subtype 1, 2 or 3 using the above protocol determined that a coating antigen of SAV1 provided the strongest signal).

### 2.11. Statistical Analysis

For RT-qPCR analysis Cq values were determined by the MX3000P cycler software. The ratios between target and reference gene (EF-1α) were calculated with the Pfaffl method [29], using primer efficiencies determined by producing a standard curve with known quantities of RNA and the average Cq of the control fish (0 dpi), which was set as 1. A general linear model (GLM) analysis was used to assess the significances of immune gene expression between different time points and the control group. GLM analysis with Poisson family data was used on pathology scores to assess the significant differences between naïve and treated fish. Time wise comparisons were calculated by least square means analyses for multiple comparisons (lsmean package) [30]. Flow cytometry data were analyzed using a GLM. Pairwise analyses were attached by least square means analyses for multiple comparisons under the lsmean package [30]. Significant differences in ELISA data were assessed using a Kruskal–Wallis test and Dunn’s post hoc tests. All data examined were considered significant when *p* ≤ 0.05. Statistical analysis was performed using the R statistical environment (v3.6.2) [31]. Graphs were produced using GraphPad Prism (v8.1.0) (Statcon, Witzenhausen, Germany).

## 3. Results

### 3.1. Virus Load

**Figure 1:**
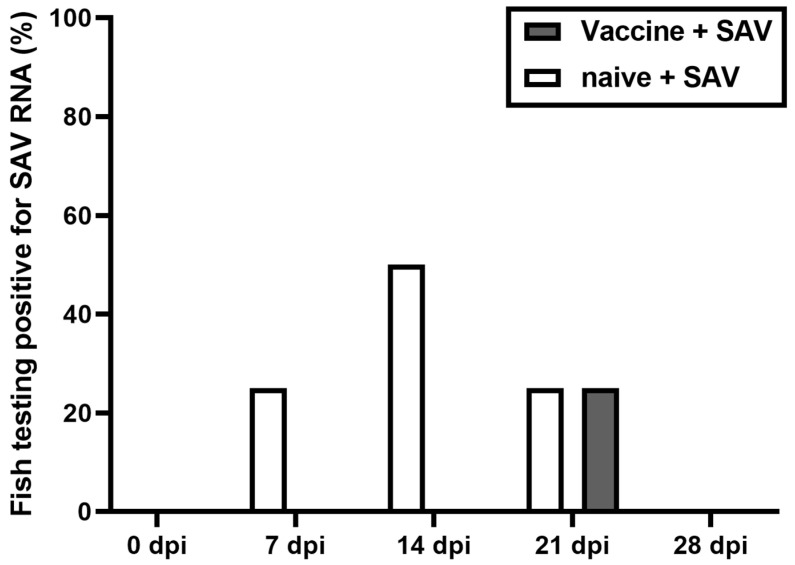
Percentage of fish exhibiting positive for salmonid alphavirus (SAV) by RT-qPCR in sera at 0, 7, 14, 21, and 28 days post challenge with SAV RNA in vaccinated and unvaccinated fish (*n* = 4).

After infection of naïve fish with SAV, the viral RNA could be first detected in sera at 7 dpi, with the peak number of fish showing viremia at 14 dpi (50%). At 28 dpi RT-qPCR could not detect the viral RNA in rainbow trout sera in this group (Figure 1). In vaccinated fish, PCR-positive fish was only observed at 21 dpi, where 25% of sera were found positive (Figure 1).

### 3.2. Pathology

**Figure 2:**
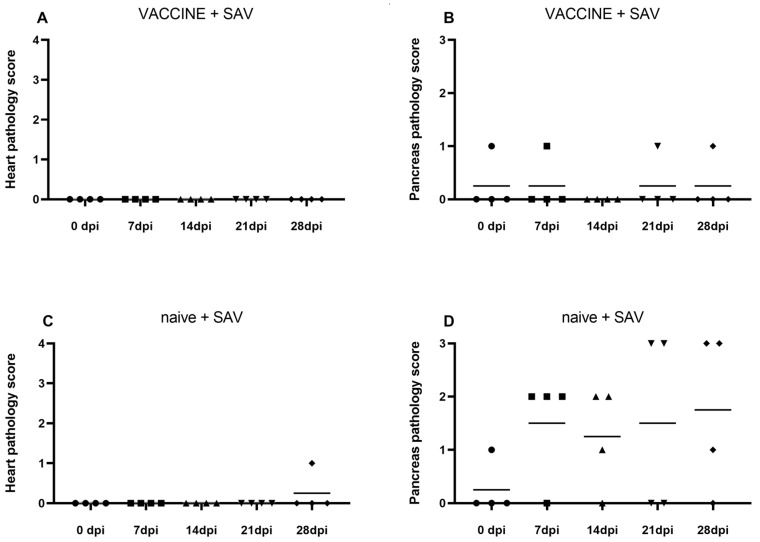
Graphs showing histological scores in heart and pancreas evaluating the impact of SAV infection at 7, 14, 21, and 28 days post injection in rainbow trout in (**A**,**B**) vaccinated and SAV challenged fish, and (**C**,**D**) naïve and SAV challenged fish over time. Heart is scored from 0–4, pancreas is scored from 0–3.

Vaccinated fish exposed to SAV developed no heart or pancreas pathology (Figure 2A,B), while only one of the naïve animals exposed to SAV developed mild heart pathology at 28 dpi (Figure 2C). A significant proportion of the naïve fish developed severe pancreas pathology, with ≥50% scoring 2–3 out of 3 (Figure 2D). The scores for pancreas pathology were found to be statistically different (*p* < 0.05) between the two groups (Figure 2B,D).

### 3.3. Cell-mediated Cytotoxicity (CMC)

Generally, PBLs exhibited the highest percentages of cytotoxicity against infected MHC-I matched target cells in comparison to splenocytes (Table 3). The highest amount of MHC-I restricted cytotoxicity was found in naïve fish infected with SAV at 21 dpi (33%) and vaccinated fish infected with SAV at 7 dpi (32%) at effector to target cell ratios of 50:1 (Table 3C,D). While cytotoxicity against infected MHC-I mismatched cells was comparably low in the same groups, this suggests an important contribution of CTLs in SAV infected fish and in fish that were vaccinated before infection.

Fish injected with adjuvant alone was the only group with remarkable cytotoxicity observed against non-infected cells. Although cytotoxicity was higher against infected cells at 14 dpv adjuvant injection, there were almost no differences between cytotoxicity against MHC-I matched and MHC-I mismatched cells, suggesting the induction of spontaneous (NK-like) cell-mediated immune responses by the adjuvant (Table 3A). Vaccinated fish exhibited either equal cytotoxicity against MHC-I matched and mismatched infected target cells (e.g., 3 dpv) or higher cytotoxicity against infected MHC-I mismatched targets when compared to infected matched target cells (e.g., at 28 dpv) in both PBLs and splenocytes (Table 3B) suggesting again a remarkable share of non-specific (NK-like) cytotoxic cells. The cytotoxicity profile of vaccinated fish that were subsequently challenged by SAV was markedly different from that observed in vaccinated fish, and naïve fish infected with SAV. Effector cells from SAV-challenged vaccinated fish were cytotoxic against infected MHC-I matched and mismatched target cells from 3 dpi until the end of the trial (28 dpi). Cytotoxicity was observed against both MHC class I matched and mismatched cells, while the highest percentages against matched targets were observed at 7 and 21 dpi by PBLs (Table 3C) suggesting a contribution of specific cytotoxic cells (CTLs). Effector cells from fish infected with SAV only, exhibited cytotoxicity against infected MHC-I matched target cells relatively late with considerable cytotoxicity at 21 dpi only, when compared to effector cells from vaccinated and challenged fish showing respective cytotoxicity already from day 3 dpi. This more rapid MHC-I restricted cytotoxicity suggests a memory response to previous vaccination (Table 3, D versus C, respectively).

### 3.4. Flow Cytometry

**Figure 3:**
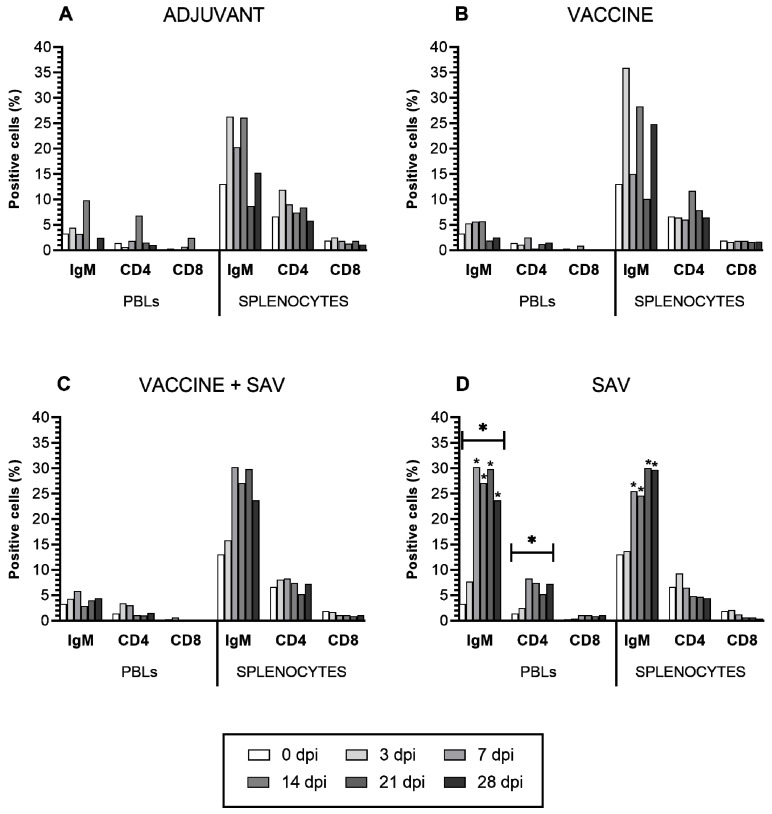
Percentages of IgM, CD4 and CD8 positive cells in PBLs and splenocytes in fish (*n* = 4, pooled) at 0, 3, 7, 14, 21, and 28 days post injection with (**A**) adjuvant, (**B**) vaccine, (**C**) SAV challenge following vaccination, and (**D**) SAV challenge. Codes: ‘*’= *p* ≤ 0.05; black star (*) with bar = comparing percentage of positive cells within a tissue to tissues of other treatment groups; black star (*) = time points compared to 0 dpi within a tissue of each treatment group.

The increase of IgM^+^ cells in splenocytes was similar in response to the adjuvant and vaccine, with the vaccinated fish showing a slightly higher percentage of IgM^+^ cells (Figure 3A,B). The profile in IgM^+^ splenocyte cells showed no clear trend, with an increase at 3 dpv (peak percentage), decrease at 7 dpv, increase at 14 dpv, decrease at 21 dpv, and increase at 28 dpv (Figure 3A,B). Vaccinated + SAV infected treatment group, and SAV infected treatment group showed a rather delayed but stable increase of IgM^+^ splenocytes from 7 dpi onwards (Figure 3C,D) compared to the previous two treatment groups. Infection of naïve fish with SAV resulted in a statistically significant (*p* ≤ 0.05) increase in IgM^+^ and CD4^+^ cells in PBLs in comparison to all other treatment groups (Figure 3D). Naïve fish exposed to SAV had a statistically significant increase in IgM^+^ cells during all days examined compared to naïve, unstimulated fish (0 dpi) in splenocytes and remarkably also in PBLs (Figure 3D).

The only significant (*p* ≤ 0.05) increase in the percentage of CD4^+^ cells was observed after SAV infection in PBLs (Figure 3D). Some small increases in percentages of positive cells were observed in splenocytes and PBLs in response to adjuvant, vaccination and vaccination followed by infection over the time course (Figure 3A–D), but levels had returned to baseline by 28 dpv/dpi. No significant changes were recorded in percentage of CD8^+^ cells in response to the treatments.

### 3.5. Gene Expression

#### 3.5.1. PBLs

**Figure 4:**
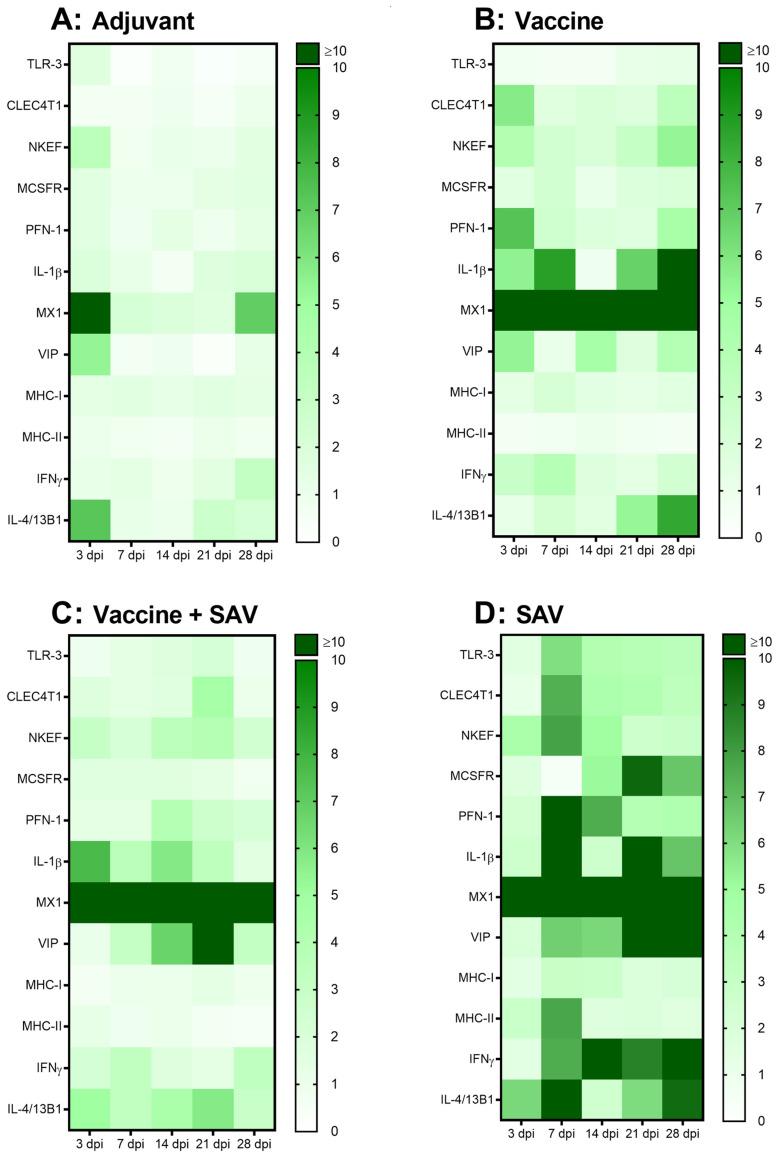
Heatmaps of RT-qPCR data showing gene expression (mean fold change, *n* = 4/treatment group/time point) in PBLs at 3, 7, 14, 21, and 28 days post injection with (**A**) adjuvant, (**B**) vaccine, (**C**) SAV challenge following vaccination, and (**D**) SAV challenge. All gene values were normalized against a housekeeping gene EF-1α followed by normalization against the average values of control group individuals.

Significant changes in gene expression were observed in response to all four treatments in PBLs (with the antiviral gene Mx the most strongly induced gene in all groups), and the largest overall changes seen in response to SAV infection (Figure 4). Fold change, SEM and *p*-values for all genes can be found in the Supplementary Materials (Table S2).

The adjuvant induced the least number of genes in PBLs where a small upregulation of MHC-II and TLR-3 occurred at 3 dpi, with the antiviral defense gene TLR-3 found to be significantly downregulated at all other time points. An upregulation (>5-fold) of NKEF, Viperin and IL-4/13 was observed at 3 dpv, however it was only significant for NKEF. The Th2 signature gene IL-4/13 was also significantly upregulated at 21 and 28 days post vaccination (Figure 4A).

In response to the vaccine, increases (>4 fold) were found at 3 dpv for CLEC4T1, NKEF, PFN-1, IL-1β and Viperin. The gene expression for the APC related gene CLEC4T1 and cytotoxicity-related genes NKEF and PFN-1 decreased to near-constitutive levels before increasing significantly again at 28 dpi. The pro-inflammatory IL-1β had prolonged increase during the time course (with the exception of 14 dpv) with highest levels of expression occurring at 28 dpv. Additionally, as seen with the adjuvant-only group IL-4/13 reached highest levels of expression at 21 and 28 dpv. MCSFR had moderate increase at 7 and 28 dpv, while an increase in the Th1 signature gene IFN-γ occurred at 3 and 7 dpv. Mx was strongly upregulated (>20-fold increase) at all time points, but significantly at 28 dpv only (Figure 4B).

Vaccinated fish, which then were challenged with SAV, had the strongest responses to the pro-inflammatory cytokine IL-1β and antiviral gene Viperin. IL-1β response was increased at 3 dpi and was maintained until 21 dpi, while Viperin expression increased steadily over time, reaching its peak at 21 dpi. Statistically significant increases were also seen with the Th2 associated gene IL-4/13 from 3 dpi to 21 dpi, and in antiviral gene TLR-3 at 21 dpi, and NKEF at 14 and 21 dpi. As in the vaccinated-only group, Mx was the most strongly upregulated gene (>20-fold increase) with statistical significance at 7, 14, and 21 dpi (Figure 4C).

Fish infected with SAV only, exhibited the most pronounced increase in gene expression of PBLs out of all treatment groups. As observed with vaccinated fish exposed to SAV the highest increases were seen in the pro-inflammatory gene IL-1β (20-fold increase) at 7 dpi, and antiviral Viperin (26-fold increase) at 21 and 28 dpi. TLR-3, NKEF and PFN-1 (associated with cytotoxicity and antiviral defense) had similar expression patterns, with pronounced statistically significant increases found at 7 dpi. Significant increases were also observed in the APC related genes MHC-II and CLEC4T1, and the Th2 signature gene IL-4/13 at 7 dpi. The Th1 signature gene IFN-γ significantly increased at 28 dpi. Mx was again the most strongly upregulated gene in response to this treatment compared to all others (>1000-fold increase) being statistically significant at 14 dpi (Figure 4D).

#### 3.5.2. Spleen

**Figure 5:**
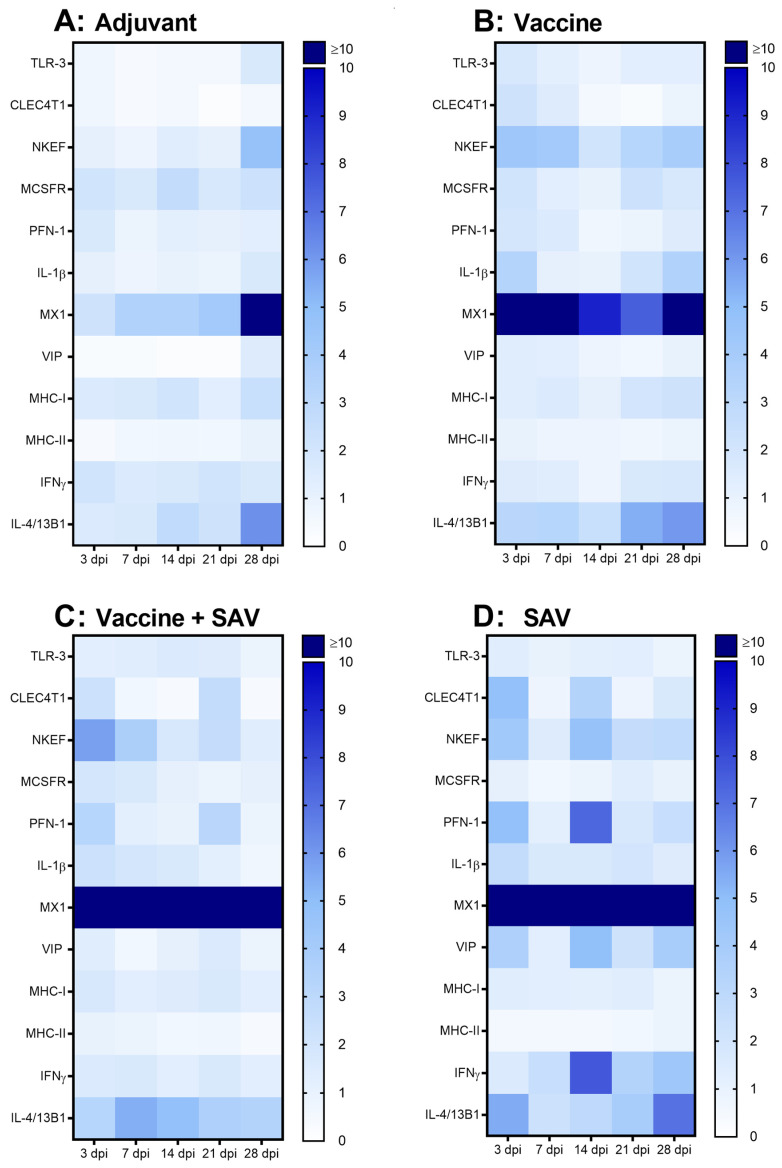
Heatmaps of RT-qPCR data showing gene expression (mean fold change, *n* = 4/treatment group/time point) in spleen at 3, 7, 14, 21, and 28 days post injection with (**A**) adjuvant, (**B**) vaccine, (**C**) SAV challenge following vaccination, and (**D**) SAV challenge. All gene values were normalized against a housekeeping gene (EF-1α) followed by normalization against the average values of control group individuals.

Significant changes in gene expression were observed in response to all four treatments in spleen. As seen in PBLs, the largest changes were in response to SAV infection and in the antiviral Mx gene (Figure 5). Fold change, SEM and *p*-values can be found in the Supplementary Materials (Table S3).

In response to the adjuvant, significant downregulation was seen in the APC-associated MHC-II and CLEC4T1 genes. The antiviral genes TLR-3 and Viperin were downregulated at 3 to 21 dpv, but slightly upregulated at 28 dpv. The largest increases in response to this treatment were found at 28 dpv with NKEF and IL-4/13 (~5-fold increase). Mx showed a statistically significant upregulation at 28 dpv (Figure 5A).

Kinetic profiles for NKEF, IL-1β, and IL-4/13 were similar in response to vaccination in spleen as PBLs. Cytotoxicity-associated NKEF and pro-inflammatory IL-1β had higher expression at 3 and 28 dpi, and Th2 associated IL-4/13 at 21 and 28 dpv. The antiviral Viperin had moderately increased in expression at 3 and 7 dpv and MCSFR at 21 dpv. Interestingly, most genes exhibited a pronounced decrease in expression at 14 dpv. Mx was the most highly upregulated gene in response to this treatment, but only statistically significant at 3 dpv (Figure 5B).

Vaccinated fish after being challenged showed an increase in CLEC4T1, NKEF, PFN-1, MCSFR, and IL-1β at 3 dpi, however the results were only significant for the pro-inflammatory cytokine IL-1β. Th2-associated IL-4/13 had the most prolonged upregulation of all genes examined, with peak expression occurring at 7 dpi. Mx was the most highly induced gene (>10-fold increase) but this change was not significant (Figure 5C).

In response to the SAV infection, although expression fold changes of 5–10 were seen for a number of genes and time points, due to large variation in fish responses, very little was found to be statistically significant. The cytotoxicity associated NKEF, PFN-1, and antiviral Viperin had similar profiles with peaks of expression at 3 and 14 dpi, which was also seen with the APC marker CLEC4T1. Pro-inflammatory IL-1β was significantly upregulated at 3 dpi, and Th2 associated IL-4/13 upregulated at 3 and 28 dpi, and the Th1 signature gene IFN-γ significantly upregulated at 14 dpi. MCSFR was found to be significantly downregulated at 7 dpi. MHC-II had low expression in response to SAV. As in all other treatment groups, Mx was the most highly induced gene in response to SAV infection (>50 fold increase) with statistical significance observed at 14 dpi (Figure 5D).

### 3.6. ELISA

**Figure 6:**
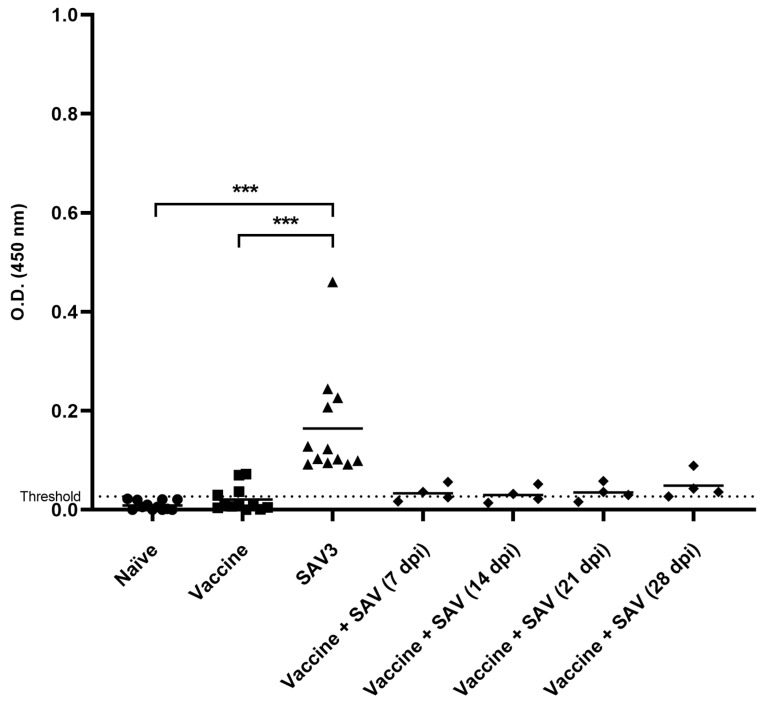
Scatter plot of specific serum antibody (IgM) levels measured by ELISA (*n* = 12). Depicted are optically density (OD) values (450 nm) of rainbow trout sera (diluted 1:100) from naïve fish, vaccinated fish (63 dpv), SAV3 challenged fish (63 dpi) and vaccinated fish at 7, 14, 21, and 28 dpi. Signif. codes: ‘***’ *p* < 0.001 comparing all treatment groups to the control. Bar = mean. Threshold = (average naïve fish OD) + (2× SD).

Specific serum antibody (IgM) levels measured by ELISA were low in vaccinated as well as in vaccinated and challenged rainbow trout (Figure 6). Although four individual fish exhibited antibody titers above the cut off at 63 dpi following vaccination with the monovalent oil-adjuvanted vaccine their titers were not significantly higher compared to naïve fish. Infection with SAV3 induced a significantly higher level of antibody titers compared to naïve, vaccinated or vaccinated and challenged fish, with high individual variability. Sera from fish that were challenged after vaccination had median optically density (OD) levels in ELISA marginally higher than sera from vaccinated fish; however, the results were not statistically significant.

## 4. Discussion

Injectable oil-adjuvanted vaccines have remained the industry standard for aquaculture since their introduction. While vaccines against SAV have reached the market in the UK and Norway [32], they do not provide complete protection in the field [18]. The lack of a cell-mediated response to the vaccine (particularly the expansion of virus-specific CTLs) could be a reason for sub-optimal efficacy [10]. Therefore, fundamental immune mechanisms after vaccination and SAV infection were examined in this paper. Our results show that while vaccination itself does not trigger a strong cytotoxic or humoral response, it will result in significantly higher and faster specific cytotoxicity following challenge, alongside reduced viral titers and pathology. An examination of cellular markers after vaccination and infection indicates that the initial innate response triggered by the vaccine could play an important role in steering adaptive mechanisms.

The co-administration of the inactivated viral antigen with an oil-adjuvant induced an effective anti-viral response. However, injection of the adjuvant alone (without antigen) was also found to trigger anti-viral effects, both on the transcriptional and the functional levels, which could be linked to the induced inflammatory environment [33]. TLRs, Mx, and Viperin (first described as Vig-1) are part of the innate anti-viral defense mechanism in fish (reviewed by [34]). TLR3 is induced by double-stranded RNA and triggers the production of type I IFNs which, in turn, induces antiviral Mx [35]. Thus, Mx can also be used as an indirect measure of viremia [36] in Atlantic salmon. Importantly, many TLRs including TLR3 tend to induce a Th1 response through type I IFN in mammals [37]. Human TLR3 has also been shown to induce Viperin [38]. According to work by [39,40] a massive induction of the Mx gene occurs in response to SAV infection. In this study, we found induction of TLR3 and Mx in PBLs correlated with serum viremia and expression of Viperin, supporting work by [41] suggesting that TLR pathways are involved in SAV recognition and signaling in salmonids.

On a functional level, salmonids have cellular immune functions resembling their mammalian counterparts, which are induced by, e.g., viruses [10]. Cell-mediated cytotoxicity is an important defense mechanism against virus-infected cells in fish [13,42,43]. It can include innate cells such as non-specific cytotoxic cells (NCC), natural killer (NK) cells, neutrophils, and macrophages which react spontaneously and immediately; and CD8^+^ cytotoxic T-cells (CTL) which require activation through MHC-I presentation [11]. As the secondary CMC response has been found to be stronger than the primary response, it implies the presence of memory CTLs [44,45]. After SAV infection, CD8^+^ T-cells and mRNA appeared to be associated with pathology and virus levels in Atlantic salmon [40,46] suggesting that CTL responses are involved in protection against this virus. However very little work has been done on CTL function, particularly cytotoxic responses induced by vaccination in fish. To date, MHC-I restricted antiviral CMC was shown to occur in ginbuna crucian carp after oral vaccination [47], and in rainbow trout after DNA vaccination [48]. To study such CMC responses, it is crucial to establish corresponding experimental systems. We observed that in contrast to Atlantic salmon, rainbow trout developed only mild clinical symptoms of pancreas/sleeping disease when infected with SAV with no mortality, however viral kinetics and pathology were found to be broadly similar. Widespread systemic immune responses post-infection coupled with homozygous clonal rainbow trout effector cell donors and MHC class I-matched and -mismatched SAV infectible target cells led to the successful development of a model for studying SAV-specific CMC in salmonids.

Following infection of rainbow trout with SAV, our results show that while some cytotoxicity against MHC-I mismatched target cells occurred, overall the response was restricted to the MHC-I matched cells, occurring one week post peak viral load. This matches work by [45] in which peak specific cytotoxic activity occurred after viral titers in infected fish had been reduced suggesting that clearance of pathogen after infection is very inefficient. In this study, vaccination induced an early and primarily innate cytotoxic response, which appears to be an adjuvant effect, as fish injected with adjuvant exhibited a similar cytotoxic response against MHC-I mismatched cells. Despite the transitory innate response induced by the vaccine, it presumably resulted in immune memory (indicated by the reduced virus load and pathology after challenge, as well as by the earlier onset of MHC-I restricted CMC) when compared to infected unvaccinated fish. However, very little changes in percentage of CD8^+^ cells and MHC-I mRNA expression were observed in our study, which has also been described previously [40,46,49]. Nevertheless, it has been reported that gene expression linked to the MHC-I antigen presentation can be stimulated by type I IFN during *in vitro* SAV infection [50]. Overall, we can conclude that (for SAV) vaccination is not enough to examine vaccine-induced immune responses and that subsequent challenge is needed to efficiently trigger those responses.

NK cells kill virus-infected cells independently from MHC class I compatibility [51]. Thus, the cytotoxicity induced here can partially be attributed to NK-like cells as has previously been described in rainbow trout [15,48,52]. Supporting this, an upregulation of NKEF was seen here after vaccination, SAV infection, and infection of vaccinated fish, with vaccination inducing strong upregulation from 3 days post injection. If some immune memory can also be attributed to NK cells is a matter of speculation, but cannot be totally ruled out since recent mammalian studies attribute NK cells as even having immune memory and executing adaptive immune functions [53,54].

The most important molecule for the specific recognition of infected antigen presenting cells (APCs) is the TCR which is present on T-helper cells and CTLs. Supporting (and at the same time signature) molecules for the binding of TLRs to the MHC/peptide complex are CD4 and CD8, respectively [25,55]. CD4^+^ cells differentiate into various subtypes following peptide presentation by MHC class II molecules and exposure to cytokines. Th1 responses are characterized by activation of cell-mediated immunity, while Th2 responses result in B-cell activation and antibody production [56]. We found a significant increase in the number of CD4^+^ cells in PBLs only following SAV infection, which corresponded to a transitory spike in MHC-II gene expression. Interestingly, significant changes in CD4^+^ cells were not observed in response to the other treatments examined, which was matched by a general trend of suppressed expression of the MHC-II gene. Fish infected with virus tend to show a skewed Th1 response [55], and IFN-γ is regarded as the typical Th1 cytokine [57] which has shown potent anti-viral activity against SAV [58]. A large upregulation of the Th1 signature gene IFN-γ was seen in response to SAV infection in our study, but only a small upregulation was seen in response to vaccination at early time points in PBLs. As work by [59,60,61] showed that immunization of salmonids with anti-viral DNA vaccines and a monovalent oil-adjuvanted SAV vaccine resulted in little modulation of IFN-γ at early time points in spleen, it suggests that while the Th1 pathway plays a role after infection, its role post-vaccination seems to be less critical.

It is thought that the induction of one Th response will suppress the other. Salmonid IL-4/13 is believed to trigger the development of Th2 cells and to increase the number of IgM secreting B-cells [62]. Notably, especially in splenocytes, IL-4/13 gene expression was significantly upregulated in all treatment groups while IFN-γ was only significantly upregulated after infection of unvaccinated fish. In the latter group, the time points of upregulation for both genes were somehow negatively correlated supporting the negative feedback between these two cytokines. The adjuvant and vaccine induced an upregulation of IL-4/13 at later time points, and virus infection in vaccinated and naïve fish resulted in a prolonged upregulation starting at 3 dpi.

Secreted IgM specific for viral proteins along with virus-neutralizing activities in serum have been shown to develop in salmon after infection with SAV [61,63,64,65,66] which our results support. However, despite the implication that protective antibody mediated immunity and an efficient adaptive response is involved in SAV infection [60], we found vaccination alone did not result in an increase in antibody titers compared to naïve fish and that only a viral challenge triggered a noteworthy antibody production. An increase in IgM^+^ cells occurred rapidly in response to all treatments, with percentages of these cells fluctuating over time in response to adjuvant or vaccine, suggesting B-cell (and presumably IgM-bearing monocyte) movement to the site of infection or immune response. However, the antigen specificity of surface IgM on these cells remains unknown.

It is known that pro-inflammatory cytokines are critical to the efficiency of the innate and subsequent adaptive responses [67], although it is currently unclear if pro-inflammatory factors are essential to bridge the later adaptive response, or if they have only a direct function. Inflammatory responses are characterized by the systemic release of specific cytokines (including IL-1β) and migration of cells to the site of inflammation. Tissue inflammation is a characteristic of SAV infection [65] and a DNA vaccine against VHSV has been found to result in the recruitment of inflammatory cells to the site of injection [48]. In the present work, vaccination against SAV induced early (from 3 dpi) and continuous upregulation of IL-1β, and this correlated with increases of IFN-γ, NKEF, and PFN-1 gene expressions. Interestingly, naïve fish infected with SAV only, also showed an increase of the same markers but at a later time point (7 dpi), (which corresponds to the time when SAV is first detected in sera). Since injection with the adjuvant only did not result in early upregulation of these genes, it supports the importance of Th1 activation for cytotoxic responses. As [68] found IL-1β peptides co-administered with a virus in trout was capable of increasing protection, this early increase in pro-inflammatory signals seen here could be an initial key component of the efficient anti-viral response.

Studies on oil adjuvants in fish have described their ability to depot antigens at the site of injection [69,70], which is thought to support sustained and robust pro-inflammatory responses and APC recruitment [71,72]. APCs (primarily macrophages, but also dendritic cells, mast cells, and B-cells) play an important role in the transition of innate immunity to adaptive immunity through phagocytosis and cell signaling (reviewed in [43]). Generally, MHC-II expression was found to be slightly but consistently downregulated in response to all treatments over time in our study. Interestingly, this did not correspond with a decrease in CD4^+^ cells. In this paper, we also used the genes for C-type lectin (CLEC) domain family 4-T1 (a rainbow trout transmembrane protein thought to be closely related to the well-characterized CLEC4 family protein CD209/DC-SIGN) [73] to examine DC responses and macrophage colony stimulating factor receptor (MCSFR) to examine macrophage responses. Supporting previous work by [74] which found CLEC4T1 and MHC-II expressed on a distinct cell population in rainbow trout following i.p. vaccination, our study found that this putative dendritic marker to be much more highly modulated than the macrophage marker in response to vaccination and infection. This is contrary to some studies on mammalian particulate vaccines describing macrophages as playing a more critical role in CTL activation and protection (reviewed by [75]) than dendritic cells. However, IL-4/13 can stimulate macrophages and dendritic cells [61], and we observed an increase in mRNA expression of CLEC4T1 correlated with increases in IL-4/13.

As discussed previously, the percentage of IgM^+^ cells increased quickly after vaccination and infection in this study. While fish IgM is the main systemic Ig, the B-cells in salmonids have maintained some innate functions, like phagocytosis [76] and expression of TLRs [77]. Activation and modulation of B-cells can occur by early innate signals, both directly or via signaling to other cells. Sensitized IgM^+^ cells from ginbuna crucian carp have been shown to exhibit spontaneous cytotoxicity against allogeneic target cells. These cells were suggested to be NK-like cells which had IgM bound on their surface via FcR [78]. Together with our finding of large non-lymphocyte IgM^+^ cells (presumably monocytes with FcR-bound IgM) these results suggest that FcR bearing cells play a role in bridging the innate and adaptive anti-viral response, particularly in response to SAV and warrants future investigation.

## 5. Conclusions

The results of this paper show that rainbow trout will mount a strong CTL-mediated response against SAV infection. Injection with a mock vaccine will induce prolonged non-specific cytotoxicity, but little modulation of cell markers. Vaccination itself will not trigger a strong or specific cytotoxic response, but it will result in reduced viral titers and pathology along with significantly faster induction of virus specific cytotoxicity following infection, indicating an efficient and specific memory response upon subsequent exposure to SAV. At a transcriptional level, vaccination will lead to a similar (but earlier) induction of inflammatory responses and recruitment of innate immune cells as infection. However, if and how the non-specific responses observed in this study result in protection remain to be elucidated. As specific antibodies were found to develop in response to the virus but not vaccination, it hints that different protective mechanisms are being triggered. Future work should aim at examining the innate and adaptive qualities of teleost B-cells and NK cells in response to vaccination, and the role vaccines play in curtailing viral evasion strategies.

## Figures and Tables

**Table 1:** Treatment groups examined to compare immune responses after vaccination, challenge, and vaccination followed by challenge.

Group	Treatment *		Time Points * Sampled	Tissues Sampled	Assessment
1	adjuvant	-	0, 3, 7, 14, 21, 28 dpv	blood, spleen	CMC, flow cytometry, RT-qPCR,
2	vaccine	-	0, 3, 7, 14, 21, 28, 63 dpv	heart, pancreas, blood, spleen	histology, CMC, flow cytometry, RT-qPCR, ELISA
3	vaccine	SAV3 challenge 43 dpv	0, 3, 7, 14, 21, 28 dpi	heart, pancreas, blood, spleen	histology, virus load, CMC, flow cytometry, RT-qPCR, ELISA
4	-	SAV3 challenge	0, 3, 7, 14, 21, 28, 63 dpi	heart, pancreas, blood, spleen	histology, virus load, CMC, flow cytometry, RT-qPCR, ELISA

* dpv/dpi = days post vaccination/infection, respectively.

**Table 2:** Information on primary and secondary antibodies used for flow cytomety analysis.

Primary Antibody (Monoclonal Antibody Against Trout Molecules)	Reference	Dilution	Secondary Antibody	Dilution
CD4 (clones 4-1(6) & 4-2b)	[24]	1:50	goat anti-rat IgG ALEXA 488	1:700
CD8α (clone 7a8c)	[25]	1:50	goat anti-rat IgG ALEXA 488	1:700
IgM (clone 1.14)	[26]	1:200	goat anti-mouse IgG, IgM ALEXA 488	1:800

**Table 3:** Percentages of cytotoxicity in PBLs and splenocytes (*n* = 3) at 0, 3, 7, 14, 21, and 28 days post injection with (A) adjuvant, (B) vaccine, (C) vaccinated and SAV, and (D) naïve and SAV challenge, against infected and non-infected MHC-I matched (RTG2) and mismatched (CHSE) cells. Grey shading signifies a percentage of cytotoxicity higher than observed with the control group (0 dpi) effector cells; numbers in bold indicate percentage cytotoxicity is significantly (*p* < 0.05) different compared to effector cells from unstimulated fish.

**A**							**B**					
**ADJUVANT**							**VACCINE**					
		PBLs		SPLENOCYTES					PBLs		SPLENOCYTES	
		MHC-I mismatched	MHC-I matched	MHC-I mismatched	MHC-I matched				MHC-I mismatched	MHC-I matched	MHC-I mismatched	MHC-I matched
0 dpv	non-infected target cells	0	0	0	0		0 dpv	non-infected target cells	0	0	0	0
3 dpv		0	0	0	0		3 dpv		0	0	0	0
7 dpv		**6**	0	**7**	0		7 dpv		0	0	0	0
14 dpv		**8**	**14**	**17**	**11**		14 dpv		0	0	0	0
21 dpv		4	0	4	0		21 dpv		6	0	9	0
28 dpv		0	0	0	0		28 dpv		0	0	0	0
0 dpv	infected target cells	4	9	0	5		0 dpv	infected target cells	4	9	0	5
3 dpv		0	12	0	10		3 dpv		14	13	11	13
7 dpv		8	8	**10**	10		7 dpv		5	3	**11**	0
14 dpv		**21**	**24**	**19**	9		14 dpv		0	**7**	0	0
21 dpv		6	4	8	0		21 dpv		0	0	4	0
28 dpv		0	0	0	0		28 dpv		24	0	14	0
												
C							D					
**VACCINE + SAV**							**naïve + SAV**					
		PBLs		SPLEENOCYTES					PBLs		SPLEENOCYTES	
		MHC-I mismatched	MHC-I matched	MHC-I mismatched	MHC-I matched				MHC-I mismatched	MHC-I matched	MHC-I mismatched	MHC-I matched
0 dpi	non-infected target cells	0	0	0	0		0 dpi	non-infected target cells	0	0	0	0
3 dpi		0	0	0	0		3 dpi		0	0	0	0
7 dpi		0	**4**	0	0		7 dpi		0	0	0	0
14 dpi		0	0	0	0		14 dpi		0	0	0	0
21 dpi		0	0	0	0		21 dpi		0	0	0	0
28 dpi		0	0	0	0		28 dpi		0	0	0	0
0 dpi	infected target cells	4	9	0	5		0 dpi	infected target cells	4	9	0	5
3 dpi		0	12	3	8		3 dpi		0	0	0	0
7 dpi		**17**	**32**	15	**19**		7 dpi		0	13	5	3
14 dpi		**18**	14	**17**	10		14 dpi		0	5	1	3
21 dpi		0	**26**	4	8		21 dpi		9	**33**	**11**	**27**
28 dpi		9	10	8	9		28 dpi		3	4	0	0

## Supplementary Materials

The following are available online at https://www.mdpi.com/2076-393X/8/4/725/s1, Table S1: Gene names, primer sequences and references used for RT-qPCR analysis [22,61,73,79,80,81,82,83,84,85,86], Table S2: RT-qPCR average fold change, SEM & *p*-values for spleen, Table S3: RT-qPCR average fold change, SEM & *p*-values for PBLs.

## Appendix A

The LDH release from killed target cells was compared to the maximum amount of LDH obtained from the same number of artificially lysed target cells. A flat-bottomed 96-wellplate comprised wells with different control reactions that assayed (1) Target cell spontaneous release (*TSR*): infected or uninfected CHSE or RTG2 cells + MM; (2) Target cell maximum release (*TMR)*: infected or uninfected CHSE or RTG2 cells + MM + Triton X-100 (Sigma-Aldrich); (3) Effector cell spontaneous release (*ESR*): PBLs or splenocytes + MM; (4) Medium background (MM): Medium alone; and (5) Experimental wells (*EXP*): Wells containing a constant number of infected or uninfected target cells (CHSE or RTG2) and a varying number of effector cells at different ratios (E:T) from 1:100 to 1:12.5.

Experiments consisting of PBLs or splenocytes of fish from the four treatment groups (Table 1) were added to non-infected and infected MHC-I matched (RTG2) and MHC-I mismatched (CHSE) target cells in triplicate. The percentage of specific *% cytotoxicity* was calculated using the following formula.

(A1) $$\%\;cytotoxicity=\frac{\left(EXP-ESR\right)-TSR}{TMR-TSR}\;\times \;100\%$$

The LDH-release assay has an advantage over the ^51^Cr cytotoxicity assay because no radioactive material needs to be handled. However, the LDH-release assay sometimes suffers from high background LDH release from effector cells especially at very high E:T cell ratios. An E:T ratio of 50:1 was the best compromise between good specific release and moderate background release. Thus, all CMC levels shown in the results section are generated at E:T ratios of 50:1.
