# Supplementary material for: Cellular Immune Responses in Rainbow Trout (Onchorhynchus mykiss) Following Vaccination and Challenge Against Salmonid Alphavirus (SAV)
Source: Vaccines (Basel). 2020 Dec 2;8(4):725. doi: 10.3390/vaccines8040725 (PMC7761581; doi:10.3390/vaccines8040725)
Supplement: Supplementary file 1 [file vaccines-08-00725-s001.pdf]

Supplementary Materials: The following are available online at www.mdpi.com/xxx/s1, **Table S1**: Gene names, primer sequences and references used for RT-qPCR analysis, **Table S2**: RT-qPCR average fold change, SEM & p-values for spleen, **Table S3**: RT-qPCR average fold change, SEM & p-values for PBLs.

Table S1: Gene names, primer sequences and references used for RT-qPCR analysis.

Abbreviation	Gene name	Forward primer (5' to 3')	Reverse primer (5' to 3')	Reference	Acc. No.
EF-1α	Elongation factor 1 alpha	CAAGGATATCCGTCGTGGCA	ACAGCGAAACGACCAAGAGG	[61]	AF498320
TLR-3	Toll-like receptor 3	AGCCCTTTGCTGCCTTACAGAG	GTCTTCAGGTCATTTTTGGACACG	[79]	AY883999
CLEC4T1 / CD209	C-Type Lectin 4 domain family 4 (trout-1) / Cluster of Differentiation 209	CACCACTGACCACAGCGAATTG	GAACATTTCTCATCATTCCACCACC	[73]	FN667662
NKEF	Natural Killer Cell Enhancement Factor	AGTAGAGGCAAAGTGAAGATGGC	AGGTGAAGTCCAGCGGGTAG	[80]	U27125
MCSFR-1a	Macrophage Colony Stimulating Factor Receptor 1a	CCAGACTTTGCACCTCCAGAGATGTATAC	GTTCTGGTGCTGCTGGGACG	[81]	AJ417832
PFN-1	Perforin 1	CTTTGGCACGCATTACATTACCA	AGCTGGCCTTGCTCTCTGTCTTA	[82]	AM29521
IL-1β	Interleukin 1 Beta-1	CCTGGAGCATCATGGCGTG	GCTGGAGAGTGCTGTGGAAGAACATATAG	[83]	AJ004821
Mx-1	Interferon-induced GTP-binding protein Mx-1	ATGCCACCCTACAGGAGATGAT	TAACTTCTATTACATTTACTATGCAA	[84]	U30253
VIP	Viperin / rsad2 / vig-1	GCAACTCCA AGCAGTGTCAA	AAACCTCTCTTTGCTTCCTCAA	[85]	AF076620
MHC-IIβ	Major Histocompatibility Complex 2 Beta chain	TCAGATTCAACAGCACTGTGGGGAG	CTTCTTCTTGTAGTAGATCAGTCCTGCT	[61]	**
MHC-I	Major Histocompatibility Complex 1	TGCATTGAKTGGCTDAAGAAGTAT	TCCAAATGACGACCCCAACAAC	[61]	**
IFN-γ1/2	Interferon Gamma 1/2	GCTGTTCAACGGAAAACCTGTTT	TCACTGTCCTCAAACGTG	[86]	AJ616215
IL-4/13B1	Interleukin 4/13 B1	GAGATTCATCTACTGCAGAGGATCATGA	GCAGTTGGAAGGGTGAAGCTTATTGTA	[61]	HG794522
Onmy-UBA*501	Allomorph of classical MHC-I locus	CCTATTGGAGATAACATACTTCGTC	AACATACCTGTAAGAATGAGAAGT	[22]	AF441856

** Primers for the polymorphic trout MHC genes were designed from the Immuno Polymorphism Database (IPD)-MHC FISH database (http://www.ebi.ac.uk/ipd/mhc/fish/)

Table S2: RT-qPCR average fold change, SEM, and p-values for spleen. Grey shading signifies gene expression value is significantly different compared to control ($p < 0.05$).

	SPLEEN				
	3 dpi	7 dpi	14 dpi	21 dpi	28 dpi

		Fold change (avg)	SEM (avg)	p-value	Fold change (avg)	SEM (avg)	p-value	Fold change (avg)	SEM (avg)	p-value	Fold change (avg)	SEM (avg)	p-value	Fold change (avg)	SEM (avg)	p-value
naïve + ADJUVANT	TLR-3	1.7	0.3	0.172	0.2	0.0	0.037	0.7	0.4	0.071	0.3	0.0	0.061	0.5	0.1	0.355
	CLEC4T1	0.7	0.2	0.063	0.6	0.1	0.006	0.8	0.4	0.016	0.4	0.2	0.002	1.0	0.7	0.034
	NKEF	3.6	0.8	0.794	0.7	0.2	0.248	1.1	0.3	0.201	1.0	0.2	0.794	1.5	0.3	0.000
	MCSFR	1.6	0.3	0.181	0.9	0.2	0.576	0.9	0.2	0.019	1.4	0.2	0.449	1.5	0.1	0.105
	PFN-1	1.6	0.2	0.156	0.8	0.1	0.417	1.4	0.7	0.927	0.8	0.3	0.785	1.3	0.9	0.912
	IL-1β	1.9	0.5	0.870	1.2	0.5	0.374	0.6	0.2	0.625	1.7	0.6	0.625	2.0	0.8	0.124
	MX1	125.5	39.5	0.328	2.2	0.7	0.241	1.9	0.4	0.233	1.7	0.3	0.127	6.9	2.4	0.000
	VIP	5.4	1.5	0.241	0.7	0.3	0.262	0.8	0.6	0.173	0.3	0.1	0.173	1.3	0.5	0.054
	MHC-I	1.3	0.2	0.051	1.6	0.1	0.104	1.3	0.2	0.154	1.5	0.1	0.091	1.4	0.1	0.594
	MHC-II	1.0	0.2	0.259	0.7	0.1	0.133	0.6	0.1	0.025	1.0	0.1	0.627	0.8	0.1	0.008
	IFNγ	1.2	0.5	0.095	1.4	0.4	0.490	0.9	0.4	0.353	1.5	0.2	0.118	3.1	2.0	0.437
	IL-4/13B1	7.2	2.3	0.955	1.2	0.1	0.955	1.0	0.2	0.212	2.7	0.5	0.522	2.2	0.6	0.000
naïve + VACCINE	TLR-3	0.7	0.2	0.245	0.5	0.2	0.919	0.6	0.1	0.286	1.2	0.4	0.963	1.3	0.6	0.979
	CLEC4T1	5.8	2.3	0.076	1.7	0.7	0.559	2.0	1.3	0.209	1.7	0.3	0.161	3.5	1.8	0.609
	NKEF	4.0	0.8	0.000	2.3	0.6	0.000	2.1	0.8	0.152	3.0	0.1	0.019	5.3	1.5	0.001
	MCSFR	1.6	0.1	0.069	2.4	0.6	0.803	1.1	0.3	0.284	1.8	0.3	0.037	2.0	0.3	0.389
	PFN-1	7.3	1.2	0.153	2.5	1.2	0.447	1.8	1.1	0.285	1.6	0.3	0.587	4.5	2.9	0.655
	IL-1β	5.5	2.2	0.014	8.7	4.5	0.945	0.8	0.2	0.918	6.8	2.7	0.285	13.1	5.0	0.008
	MX1	42.6	36.5	0.006	34.2	25.8	0.122	31.2	15.7	0.311	16.4	4.9	0.457	61.3	24.5	0.133
	VIP	5.4	2.5	0.011	1.1	0.6	0.024	4.6	3.4	0.943	1.8	0.7	0.602	4.0	1.3	0.325
	MHC-I	1.4	0.2	0.439	2.1	0.4	0.264	1.5	0.1	0.362	1.3	0.1	0.236	1.6	0.2	0.439
	MHC-II	0.6	0.0	0.395	0.8	0.1	0.263	1.0	0.3	0.871	0.7	0.1	0.058	0.6	0.1	0.010
	IFNγ	2.9	1.1	0.651	3.8	0.9	0.789	1.8	0.3	0.402	1.4	0.7	0.432	2.3	0.7	0.264
	IL-4/13B1	1.2	0.4	0.122	2.3	0.4	0.089	1.5	0.3	0.367	5.3	1.4	0.001	8.4	1.5	0.000

VACCINE + SAV	TLR-3	0.8	0.1	0.913	1.4	0.2	0.861	1.7	0.3	0.449	2.2	0.7	0.572	0.8	0.1	0.424
	CLEC4T1	1.8	0.4	0.433	1.3	0.5	0.656	1.6	0.9	0.582	4.6	4.1	0.334	1.0	0.5	0.582
	NKEF	3.0	0.1	0.001	2.1	0.5	0.042	3.6	1.7	0.477	3.9	1.5	0.149	2.3	0.8	0.759
	MCSFR	1.6	0.2	0.116	1.6	0.2	0.297	1.7	0.3	0.332	1.4	0.2	0.108	0.7	0.1	0.281
	PFN-1	1.3	0.3	0.195	1.4	0.6	1.000	3.9	2.9	0.967	2.7	1.6	0.215	2.2	1.1	0.903
	IL-1β	7.7	3.6	0.014	3.7	2.2	0.089	5.8	4.9	0.211	3.4	1.8	0.808	1.5	0.3	0.369
	MX1	15.4	6.8	0.817	51.8	13.6	0.545	72.6	18.7	0.524	46.0	21.1	0.053	22.3	5.4	0.740
	VIP	1.2	0.3	0.356	3.0	0.8	0.897	6.7	2.1	0.587	11.8	5.9	0.172	3.2	0.7	0.736
	MHC-I	0.7	0.1	0.458	1.0	0.1	0.337	1.0	0.2	0.127	1.4	0.2	0.146	0.9	0.1	0.054
	MHC-II	1.3	0.0	0.064	0.8	0.1	0.592	1.0	0.3	0.064	0.5	0.1	0.146	0.5	0.0	0.166
	IFNγ	2.2	0.6	0.546	3.2	0.9	0.511	1.7	0.4	0.880	1.4	0.3	0.48	3.3	2.4	0.869
	IL-4/13B1	5.0	2.0	0.208	3.3	1.6	0.005	4.4	0.9	0.014	5.8	0.5	0.114	2.9	0.4	0.151
naïve + SAV	TLR-3	1.5	0.5	0.884	5.9	3.5	0.610	4.0	1.8	0.985	3.7	1.8	0.884	3.6	1.6	0.572
	CLEC4T1	1.2	0.3	0.163	7.4	3.0	0.894	4.3	3.3	0.373	4.1	1.9	0.931	3.4	1.6	0.914
	NKEF	4.4	1.5	0.140	7.8	2.9	0.817	4.9	3.3	0.089	2.7	1.0	0.459	2.9	1.0	0.414
	MCSFR	1.8	0.5	0.406	0.5	0.4	0.046	5.2	2.6	0.195	9.6	8.7	0.897	6.8	4.4	0.308
	PFN-1	2.3	0.4	0.363	11.1	5.9	0.980	7.5	6.1	0.091	3.9	1.7	0.862	4.1	1.6	0.795
	IL-1β	2.6	0.6	0.030	23.2	16.5	0.416	2.6	0.5	0.374	17.7	11.1	0.206	6.8	1.6	0.534
	MX1	25.3	9.3	0.500	51.0	30.3	0.215	2389.3	1925.2	0.018	1546.3	767.5	0.362	3618.4	2217.5	0.064
	VIP	2.0	0.3	0.288	6.5	3.4	0.842	6.2	3.3	0.111	26.6	12.5	0.527	25.0	13.8	0.218
	MHC-I	1.4	0.7	0.079	2.9	1.7	0.091	2.8	1.1	0.079	1.9	0.4	0.154	2.1	0.6	0.347
	MHC-II	2.8	1.7	0.166	7.7	5.1	0.521	1.8	0.2	0.857	1.9	0.2	0.11	1.7	0.1	0.607
	IFNγ	1.5	0.5	0.906	7.5	4.4	0.681	15.5	6.5	0.038	8.8	4.9	0.468	17.6	11.5	0.296
	IL-4/13B1	6.2	1.5	0.036	22.4	11.4	0.686	2.5	0.3	0.478	6.1	0.3	0.199	9.5	4.2	0.005

Table S3: RT-qPCR average fold change, SEM, and p-values for PBLs. Grey shading signifies gene expression value is significantly different compared to control (p < 0.05).

		PBLs														
		3 dpi			7 dpi			14 dpi			21 dpi			28 dpi		
		Fold change (avg)	SEM (avg)	p-value	Fold change (avg)	SEM (avg)	p-value	Fold change (avg)	SEM (avg)	p-value	Fold change (avg)	SEM (avg)	p-value	Fold change (avg)	SEM (avg)	p-value
naïve + ADJUVANT	TLR-3	0.7	0.1	0.006	0.4	0.1	0.000	0.5	0.1	0.004	0.5	0.1	0.001	1.7	0.4	0.012
	CLEC4T1	0.7	0.2	0.081	0.4	0.1	0.467	0.5	0.2	0.682	0.2	0.1	0.385	0.5	0.1	0.771
	NKEF	1.1	0.0	0.023	0.8	0.1	0.567	1.4	0.2	0.998	1.1	0.2	0.824	4.7	0.4	0.590
	MCSFR	2.1	0.2	0.200	1.7	0.2	0.634	2.7	0.7	0.683	1.8	0.2	0.243	2.3	0.6	0.079
	PFN-1	1.7	0.2	0.056	0.9	0.1	0.710	1.2	0.3	0.941	1.1	0.2	0.694	1.3	0.2	0.918
	IL-1β	1.1	0.2	0.357	0.8	0.3	0.975	1.0	0.3	0.459	0.9	0.1	0.462	1.7	0.5	0.228
	MX1	2.2	0.5	0.793	3.5	1.4	0.194	3.5	1.8	0.189	4.1	1.1	0.111	17.2	1.3	0.180
	VIP	0.3	0.1	0.096	0.3	0.2	0.561	0.2	0.0	0.760	0.2	0.1	0.239	1.5	0.7	0.791
	MHC-I	1.6	0.2	0.109	1.7	0.3	0.118	2.1	0.4	0.123	1.3	0.5	0.403	2.4	0.3	0.159
	MHC-II	0.4	0.1	0.040	0.6	0.1	0.066	0.7	0.1	0.519	0.6	0.0	0.073	1.0	0.1	0.270
	IFNγ	2.1	0.3	0.775	1.6	0.3	0.939	1.7	0.4	0.597	2.1	0.5	0.998	1.7	0.1	0.143
	IL-4/13B1	1.6	0.2	0.703	1.7	0.6	0.928	2.8	0.5	0.595	2.2	0.2	0.006	6.2	2.0	0.048
naïve + VACCINE	TLR-3	1.8	0.2	0.362	1.2	0.2	0.173	0.8	0.2	0.302	1.3	0.1	0.783	1.3	0.3	0.676
	CLEC4T1	2.2	0.9	0.005	1.5	0.2	0.760	0.5	0.2	0.618	0.3	0.1	0.716	0.9	0.3	0.098
	NKEF	4.3	0.6	0.011	4.1	0.6	0.175	2.1	0.6	0.359	3.2	0.3	0.042	3.9	0.8	0.000
	MCSFR	2.1	0.2	0.249	1.3	0.4	0.003	1.0	0.1	0.913	2.3	0.3	0.057	1.8	0.3	0.032
	PFN-1	1.9	0.5	0.005	1.6	0.4	0.561	0.7	0.1	0.861	0.9	0.1	0.947	1.5	0.4	0.088
	IL-1β	3.3	0.6	0.285	1.1	0.2	0.046	1.0	0.3	0.924	2.1	1.3	0.131	3.5	1.0	0.003

	MX1	24.3	10.1	0.127	13.3	5.6	0.185	9.1	1.7	0.279	7.5	1.8	0.591	13.0	6.3	0.016
	VIP	1.4	0.2	0.023	1.3	0.1	0.993	0.8	0.1	0.056	0.6	0.1	0.656	1.0	0.2	0.083
	MHC-I	1.4	0.4	0.121	1.6	0.3	0.178	1.1	0.1	0.398	2.0	0.2	0.136	2.2	0.5	0.077
	MHC-II	1.0	0.1	0.341	0.8	0.1	0.003	0.8	0.1	0.257	0.7	0.1	0.467	0.9	0.2	0.126
	IFNγ	1.5	0.3	0.177	1.4	0.4	0.016	0.8	0.2	0.779	1.7	0.3	0.944	1.8	0.5	0.344
	IL-4/13B1	3.1	0.3	0.977	3.2	0.7	0.284	2.4	0.5	0.766	5.4	1.3	0.001	6.0	1.0	0.000
	TLR-3	1.3	0.3	0.163	1.4	0.3	0.462	1.6	0.2	0.078	1.5	0.5	0.010	0.9	0.1	0.401
	CLEC4T1	2.3	1.9	0.949	0.6	0.2	0.959	0.4	0.1	0.805	2.7	2.2	0.051	0.4	0.1	0.878
	NKEF	5.8	2.1	0.066	3.7	0.3	0.363	1.8	0.2	0.031	2.6	0.7	0.021	1.4	0.2	0.271
	MCSFR	1.9	0.2	0.003	1.7	0.3	0.093	1.1	0.1	0.057	0.9	0.1	0.306	1.1	0.1	0.301
	PFN-1	3.2	2.2	0.926	1.2	0.3	0.957	1.0	0.1	0.160	3.1	2.1	0.520	0.9	0.1	0.688
VACCINE + SAV	IL-1β	2.3	0.4	0.006	1.9	0.3	0.473	1.7	0.6	0.182	1.2	0.1	0.558	0.7	0.1	0.924
	MX1	11.0	2.5	0.210	26.5	7.2	0.002	27.8	12.7	0.000	10.6	2.4	0.011	15.1	2.7	0.201
	VIP	1.4	0.6	0.598	0.6	0.2	0.414	1.1	0.3	0.025	1.6	0.9	0.001	0.9	0.1	0.381
	MHC-I	1.8	0.1	0.097	1.3	0.5	0.207	1.5	0.1	0.412	1.7	0.7	0.096	1.3	0.1	0.063
	MHC-II	1.0	0.1	0.161	0.9	0.1	0.629	0.6	0.1	0.821	0.7	0.1	0.185	0.4	0.1	0.389
	IFNγ	1.6	0.0	0.564	1.7	0.3	0.192	1.3	0.3	0.871	1.7	1.0	0.925	1.3	0.2	0.167
	IL-4/13B1	3.2	0.7	0.000	5.4	1.5	0.032	4.8	1.3	0.002	3.6	0.9	0.000	3.4	0.7	0.076
	TLR-3	1.4	0.5	0.815	1.0	0.3	0.025	1.2	0.6	0.160	1.3	0.4	0.158	0.9	0.3	0.174
	CLEC4T1	4.8	4.3	0.961	0.8	0.3	0.002	3.4	2.9	0.891	0.8	0.2	0.088	1.7	1.2	0.187
	NKEF	4.2	2.9	0.033	1.5	0.2	0.000	4.7	3.1	0.709	2.6	0.8	0.265	2.8	0.8	0.221
naïve + SAV	MCSFR	1.1	0.2	0.882	0.6	0.2	0.916	0.9	0.3	0.687	1.4	0.3	0.084	1.0	0.2	0.238
	PFN-1	4.8	3.9	0.747	1.2	0.3	0.001	7.3	6.9	0.977	1.8	0.3	0.308	2.5	1.2	0.278
	IL-1β	2.7	1.0	0.856	1.7	0.4	0.017	1.7	0.3	0.869	2.0	0.4	0.044	1.5	0.5	0.476
	MX1	34.7	19.9	0.988	63.5	26.1	0.975	126.0	72.6	0.123	46.6	35.1	0.263	96.4	44.1	0.014

	VIP	3.6	3.1	0.915	1.3	0.4	0.592	4.9	3.0	0.613	2.2	1.4	0.010	3.9	1.9	0.015
	MHC-I	1.4	0.3	0.967	1.3	0.1	0.122	1.2	0.3	0.729	1.4	0.1	0.559	0.9	0.3	0.441
	MHC-II	0.5	0.0	0.374	0.5	0.1	0.005	0.5	0.1	0.805	0.6	0.2	0.663	0.9	0.2	0.760
	IFNγ	1.6	0.3	0.996	2.5	2.1	0.424	7.7	5.6	0.164	3.4	1.6	0.289	4.3	1.7	0.023
	IL-4/13B1	5.5	2.0	0.263	2.3	0.6	0.000	2.9	0.5	0.783	3.9	0.8	0.275	7.0	2.5	0.072
